# EARLY COMPLICATIONS OF SURGICAL TREATMENT OF CERVICAL SPONDYLOTIC MYELOPATHY

**DOI:** 10.1590/1413-785220233104e260397

**Published:** 2023-07-31

**Authors:** RICARDO LUCCA CABARITE SAHEB, THABATA PASQUINI SOEIRA, LUCAS MORATELLI, MARIANA DEMÉTRIO DE SOUSA PONTES, CARLOS FERNANDO PEREIRA DA SILVA HERRERO

**Affiliations:** 1 Universidade de São Paulo, Faculdade de Medicina de Ribeirão Preto FMRP USP, Departamento de Ortopedia e Anestesiologia, Ribeirão Preto, SP, Brazil.; 2 Universidade de São Paulo, Faculdade de Medicina, Hospital das Clínicas, Instituto de Ortopedia e Traumatologia IOT HCFMUSP, São Paulo, SP, Brazil.

**Keywords:** Cervical Cord, Spondylosis, Spinal Cord Compression, Medula Cervical, Espondilose, Compressão da Medula Espinal

## Abstract

**Objective:**

To evaluate the early postoperative complications associated with the surgical approach of the cervical spine of patients with cervical spondylotic myelopathy (CSM), comparing the anterior surgical, the posterior surgical, and the combined approaches.

**Methods:**

This is a retrospective study based on a database with 169 patients. Demographic data, such as gender and age, and surgical data, such as surgical approach, number of segments with arthrodesis, surgical time, and complications, were evaluated. Complications were divided into major (deep surgical wound infection, intercurrence with the implant, early new compression, and heart failure) and minor (dysphagia, superficial infection, pain, urinary intercurrence, neuropraxia of the C5 root, acute confusional state, and surgical wound hematoma).

**Results:**

This included 169 patients, 57 women (33.7%) and 112 men (66.2%). Age ranged from 21 to 87 years, with a mean of 56.48 (± 11) years. Of these, 52 (30.8%) underwent the anterior approach; 111 (65.7%), the posterior approach; and 6 (3.5%), the combined approach.

**Conclusion:**

As in the literature, we evinced dysphagia, pain, and superficial infection of the surgical wound as the most frequent postoperative complications. However, it was impossible to establish a statistical relationship between the incidence of complications and surgical time, access route, and number of fixed segments. ***Level of Evidence III, Retrospective Comparative Study.***

## RESUMO

**Objetivo:**

Avaliar as complicações pós-operatórias precoces associadas à abordagem cirúrgica da coluna cervical de pacientes portadores de mielopatia cervical espondilótica (MCE), comparando a abordagem cirúrgica anterior, a abordagem cirúrgica posterior e a abordagem combinada.

**Métodos:**

Estudo retrospectivo baseado em um banco de dados com 169 pacientes. Foram avaliados dados demográficos, como gênero e idade, e dados cirúrgicos, como abordagem cirúrgica realizada, número de segmentos artrodesados, tempo cirúrgico e complicações. As complicações foram divididas em maiores (infecção profunda da ferida operatória, intercorrência com o implante, nova compressão precoce, insuficiência cardíaca) e menores (disfagia, infecção superficial, dor, intercorrência urinária, neuropraxia da raiz de C5, estado confusional agudo, hematoma de ferida operatória).

**Resultados:**

Foram incluídos 169 pacientes, sendo 57 do sexo feminino (33,7%) e 112 do masculino (66,2%). A idade variou de 21 a 87 anos, com média de 56,48 anos (*±* 11). Destes, 52 (30,8%) foram submetidos à abordagem anterior, 111 (65,7%) à abordagem posterior e 6 (3,5%) à abordagem combinada.

**Conclusão:**

Assim como na literatura, evidenciamos a disfagia, a dor e a infecção superficial da ferida operatória como as complicações pós-operatórias mais frequentes. No entanto, não foi possível estabelecer uma relação estatística da incidência de complicações com o tempo cirúrgico, a via de acesso e o número de segmentos fixados. **Nível de Evidência III, Estudo Retrospectivo Comparativo.**

## INTRODUCTION

Cervical spondylotic myelopathy (CSM) is a general term to characterize an age-related degenerative process that corresponds to a set of changes involving vertebrae, intervertebral discs, facet joints, and associated ligaments. ^(^ ^1^ A striking feature of this evolution is the formation of osteophytes, which develop from vertebral bodies in an attempt to add stability to areas with disc degeneration and hypermobility. ^(^ ^2^ Moreover, they often occur concomitantly with disc protrusion, hypertrophy of uncovertebral and facet joints, and thickening or hypertrophy of the flavum. Such factors associated with the degenerative process contribute to narrowing the vertebral canal and potentially the spinal cord. ^(^ ^3^

Cervical spondylosis typically affects several vertebral segments and estimates suggest it affects from 70% to 95% of individuals over 60 years of age asymptomatically; ^(^ ^4^ configuring an important cause of neurological dysfunction and the primary source of spinal cord dysfunction in individuals over 55 years of age. ^(^ ^5^

Surgical treatment is indicated in moderate to severe neurological symptoms or cases with worsened neurological deficits. It involves decompressing the compromised neural structures (which may be followed by surgical stabilization of the involved vertebral segments). ^(^ ^2^ Available approaches for surgical treatment consist of the anterior, posterior, or combined approaches (the latter involves both the anterior and posterior approaches). ^(^ ^6^ However, controversy remains about the best approach to surgically treat patients with cervical spondylotic myelopathy. ^(^ ^7^

Previous studies have shown the advantages and disadvantages of different approaches to the cervical spine and compared surgical complications related to each approach.^8^ Nevertheless, studies have investigated the clinical outcomes of several diseases that led to the surgical treatment of the cervical spine.^9^ Thus, this study aimed to identify the early postoperative complications associated with the surgical approach to the cervical spine in patients with CSM, comparing the anterior, posterior, and combined surgical approaches.

## METHODS

A retrospective study was conducted based on electronic clinical records of patients who underwent surgical procedures to treat CSM at the Ribeirao Preto Medical School Clinics Hospital of University of Sao Paulo (HCFMRP-USP), from 2008 to 2015. This study was approved by the Institutional Ethics Board (Ribeirao Preto Medical School Clinics Hospital of the University of Sao Paulo) under Registration number 1.575.506 (CAAE: 56419516.1.0000.5440), and all patients signed informed consent forms.

We assessed patients’ demographic data (gender, age) and surgery-related data (surgical approach, operated spine levels, duration of surgery). Male and female patients aged above 18 years with complete registration data - including gender, age, comorbidities, type of surgical procedure, and early complications - were included. Patients with incomplete registration data and previous surgery were excluded.

The procedure performed in patients undergoing the anterior approach involved performing a discectomy or corpectomy associated with the placement of an intersomatic device and fixation with a plate for decompression and arthrodesis, whereas in patients undergoing the posterior approach, the surgical procedure comprised laminectomy associated with fixation with screws of lateral mass and bars.

Complications were divided into major and minor. We included all adverse events, and complications were defined as major when the adverse event led to permanent sequelae or required additional surgical intervention. On the other hand, complications were considered minor when the adverse event neither deteriorated the clinical picture neither required additional surgical intervention. The time considered for evaluating the adverse event was 30 days from the date of surgery.

We described the data by measures of central tendency, dispersion, and frequencies. The assessment of normality of the continuous variable was obtained by the Shapiro-Wilk test. Inferential analyses were performed using Pearson’s correlation and Fisher’s exact tests to assess correlations between categorical variables and the Student’s T-test of independent samples to assess difference in means. Multivariate analysis was obtained by multinomial logistic regression. SPSS, version 24, for Windows (Armonk, NY, USA) was used for statistical analyses, assuming a significance level of 5%.

## RESULTS

**Figure 1:**
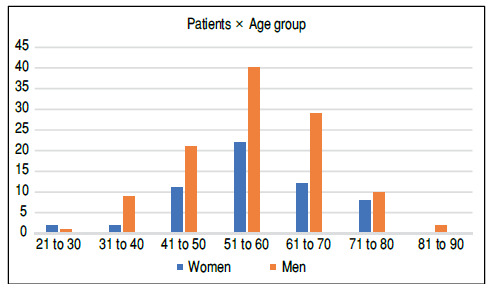
Distribution of patients according to gender and age group.

This study included 169 patients, 57 of which were women (33.7%) and 112 men (66.2%). Their age ranged from 21 to 87 years, with a mean of 56.48 years (± 11). Figure 1 shows the distribution of patients according to gender and age group.

**Figure 2:**
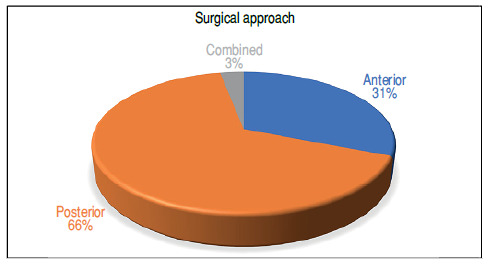
Distribution of patients according to the used approach.

Of the 169 patients included in this study who underwent surgical procedures, 52 (30.8%) underwent the anterior approach; 111 (65.7%), the posterior approach; and 6 (3.5%), the combined approach (anterior and later), as shown in Figure 2.

In patients who underwent the anterior approach, the number of fused segments ranged from 1 two 4, with 19 patients (36.53%) undergoing fixation of 1 segment; 19 patients (36.53%), of 2 segments; 11 patients (21.15%), of 3 segments; and 3 patients, (5.7%) of 4 segments. In patients who underwent the posterior approach, the number of fused segments ranged from 2 to 9 segments, with 2 patients (1.8%) undergoing fixation of 2 segments; 20 patients (18.01%), of 3 segments; 39 patients (35.13%), of 4 segments; 38 patients (34.23%), of 5 segments; 10 patients (9%), of 6 segments; 1 patient (0.9%), of 7 segments; and 1 patient (0.9%), of 9 segments. The combined approach involved fusion of 2 to 6 segments, with 2 patients (33.33%) undergoing fixation of 2 segments; 1 patient (16.66%), of 3 segments; 1 patient (16.66%), of 4 segments; 1 patient (16.66%), of 5 segments; and 1 patient (16.66%), of 6 segments.

The mean surgical time of the procedures performed by the anterior approach was 179 minutes, with a standard deviation of 53.5 minutes (ranging from 95 to 440 minutes). The surgical time of the procedures performed by the posterior approach had a mean of 224 minutes and a standard deviation of 62 minutes (ranging from 102 to 480 minutes); and the surgical time of procedures performed by the combined anterior/posterior approach had a mean of 333 minutes, with a standard deviation of 108 minutes (ranging from 138 to 463 minutes).

From a total of 169 operated patients, we found 64 complications (37.9%). Of these, 21 (12.4%) represented major complications and 43 (25.5%), minor complications. Of the major complications, 11 referred to deep surgical wound infections; five, to cardiovascular complications; four, to complications with implants, and 1 case with new early root compression. Minor complications involved12 cases of pain, nine of dysphagia, seven of superficial infection, five surgical wound hematomas, five of C5 root neuropraxia, four4 of urinary complications, and four 4 which evolved into acute confusional state.

When comparing complications regarding the number of fused levels, one group consisting of patients with up to 2 levels of fixation and another, of patients undergoing three or more levels of fixation, dysphagia was the only complication associated with the number of fused levels with statistical significance (p = 0.005) (Table 1).

**Table 1:** Complications according to the number of fused levels (N = 169).

Complication	N (N = 169)	Compl. depending on the number of levels, n (%)		p-value
		Up to 2 levels (n = 45)	3 or more (n = 124)	
Dysphagia	9 (5.3%)	6 (13.3%)	3 (2.4%)	0.005
Superficial infection	7 (4.1%)	3 (6.7%)	4 (3.2%)	0.321
Urinary Intercur.	4 (2.4%)	0 (0%)	4 (3.2%)	0.223
Pain	12 (7.1%)	6 (13.3%)	6 (4.8%)	0.057
Confusional state	4 (2.4%)	1 (2.2%)	3 (2.4%)	0.941
Hematoma	5 (3.0%)	2 (4.4%)	3 (2.4%)	0.492
C5 neuropraxia	5 (3.0%)	0 (0%)	5 (4.0%)	0.171
Deep infection*	11 (6.5%)	1 (2.2%)	10 (8.1%)	0.174
Implant intercur.*	4 (2.4%)	0 (0%)	4 (3.2%)	0.223
New compression*	1 (0.6%)	1 (2.2%)	0 (0%)	0.096
Cardiac intercur.*	5 (3.0%)	2 (4.4%)	3 (2.4%)	0.492
Death*	2 (1.2%)	0 (0%)	2 (1.6%)	0.391
*Major complic.	21 (12.4%)	4 (8.9%)	17 (13.7%)	0.401
**Total complic.**	64 (37.9%)	22 (48.9%)	42 (33.9%)	0.075

* Major complications.

When we separately evaluated complications regarding the number of fused levels in the different approaches, we found no statistically significant difference in the anterior, posterior, and combined approaches (Tables 2, 3, and 4).

**Table 2:** Complications regarding the number of fused levels in the different approaches: anterior approach.

Complication	N (N = 52)	Compl. depending on the number of levels, n (%)		p-value**
		Up to 2 levels (n = 40)	3 or more (n = 12)	
Dysphagia	9 (17.3%)	6 (15.0%)	3 (25.0%)	0.340
Superficial infection	2 (3.8%)	1 (2.5%)	1 (8.3%)	0.412
Pain	4 (7.7%)	4 (10.0%)	0 (0.0%)	0.338
Confusional state	2 (3.8%)	1 (2.5%)	1 (8.3%)	0.412
Hematoma	2 (3.8%)	2 (5.0%)	0 (0.0%)	0.588
Deep infection*	1 (1.9%)	1 (2.5%)	0 (0.0%)	0.769
Cardiac intercur.*	2 (3.8%)	2 (5.0%)	0 (0.0%)	0.588
**Major complic.**	3 (5.8%)	3 (7.5%)	0 (0.0%)	0.447
**Total complic.**	21 (40.4%)	17 (42.5%)	4 (33.3%)	0.413

* Major complications; **Fisher’s exact test.

**Table 3:** Complications regarding the number of fused levels in the different approaches: posterior approach.

Complication	N (N = 110)	Compl. depending on the number of levels, n (%)		p-value**
		Up to 2 levels (n = 2)	3 or more (n = 108)	
Superficial infection	4 (3.6%)	1 (50.0%)	3 (2.8%)	0.072
Urinary Intercur.	4 (3.6%)	0 (0.0%)	4 (3.7%)	0.928
Pain	7 (6.4%)	1 (50.0%)	6 (5.6%)	0.124
Confusional state	2 (1.8%)	0 (0.0%)	2 (1.9%)	0.964
Hematoma	3 (2.7%)	0 (0.0%)	3 (2.8%)	0.946
C5 neuropraxia	4 (3.6%)	0 (0.0%)	4 (3.7%)	0.928
Deep infection*	10 (9.1%)	0 (0.0%)	10 (9.3%)	0.826
Implant intercur.*	3 (2.7%)	0 (0.0%)	3 (2.8%)	0.946
Cardiac intercur.*	3 (2.7%)	0 (0.0%)	3 (2.8%)	0.946
Death*	2 (1.8%)	0 (0.0%)	2 (1.9%)	0.964
**Major complic.**	16 (14.5%)	0 (0.0%)	16 (14.8%)	0.729
**Total complic.**	38 (34.5%)	2 (100%)	36 (33.3%)	0.117

* Major complications; **Fisher’s exact test.

**Table 4:** Complications regarding the number of fused levels in the different approaches: combined approach.

Complication	N (N = 7)	Compl. depending on the number of levels, n (%)		p-value**
		Up to 2 levels (n = 3)	3 or more (n = 4)	
Superficial infection	1 (14.3%)	1 (33.3%)	0 (0.0%)	0.429
Pain	1 (14.3%)	1 (33.3%)	0 (0.0%)	0.429
C5 neuropraxia	1 (14.3%)	0 (0.0%)	1 (25.0%)	0.571
Implant intercur.*	1 (14.3%)	0 (0.0%)	1 (25.0%)	0.571
New compression*	1 (14.3%)	1 (33.3%)	0 (0.0%)	0.429
**Major complic.**	2 (28.6%)	1 (33.3%)	1 (25.0%)	0.714
**Total complic.**	5 (71.4%)	3 (100%)	2 (50.0%)	0.286

* Major complications; **Fisher’s exact test.

By correlating surgical time with the presence or absence of complications, we found a statistically significant difference in patients with superficial surgical wound infections (p = 0.014), complications with implants (p = 0), and between total complications (p = 0.005) (Table 5).

**Table 5:** Duration of the procedure according to the occurrence of complications (N = 169).

Complication	Occurrence of complications		Mean difference ± Standard error of the diff.	p-value
	Yes Mean ± SD	No Mean ± SD		
Dysphagia	187.7 ± 43.2	217.6 ± 71.0	−29.9 ± 24.0	0.214
Superficial infection	279.3 ± 89.6	213.2 ± 68.1	66.0 ± 26.6	0.014
Urinary Intercur.	257.8 ± 56.3	215.0 ± 70.2	42.8 ± 35.4	0.229
Pain	270.4 ± 102.3	211.8 ± 65.6	58.6 ± 30.0	0.075
Confusional state	221.0 ± 85.6	215.9 ± 70.0	5.1 ± 35.6	0.885
Hematoma	207.0 ± 54.5	216.3 ± 70.6	−9.3 ± 31.9	0.772
C5 neuropraxia	248.0 ± 92.6	215.0 ± 69.4	33.0 ± 31.8	0.301
Deep infection*	196.6 ± 45.4	217.3 ± 71.4	−20.8 ± 21.9	0.343
Implant intercur.*	345.0 ± 91.5	212.9 ± 66.8	132.1 ± 34.1	0.000
New compression*	138.0 ± 0.0	216.45 ± 70.0	−78.5 ± 70.2	0.266
Cardiac intercur.*	209.6 ± 24.5	216.2 ± 71.0	−6.6 ± 31.9	0.837
Death*	197.5 ± 24.8	216.2 ± 70.4	−18.7 ± 50.0	0.709
**Major complic.**	225.1 ± 78.5	214.7 ± 69.0	10.5 ± 16.4	0.524
**Total complic.**	235.4 ± 82.0	204.1 ± 59.0	31.3 ± 10.9	0.005

* Major complications.

When we separately evaluated surgical time according to complications in the different approaches, we found no statistically significant difference in the anterior approach. (Table 6) Regarding patients who underwent the posterior approach, we observed a statistically highermean duration of surgery in cases with pain (p = 0.000) and complications with implants (p = 0.016) (Table 7). And regarding patients who had undergone surgery by the combined approach, we found a statistical difference when we evaluated one major complication: early compression (p = 0.014) (Table 8).

**Table 6:** Duration of the procedure depending on the occurrence of complications stratified by surgical approach: anterior approach.

Complication	Occurrence of complications		Mean difference ± Standard error of the diff.	p-value
	Yes Mean ± SD	No Mean ± SD		
Dysphagia	187.7 ± 43.2	181.2 ± 57.1	6.4 ± 20.2	0.751
Superficial infection	315.0 ± 177.0	177.0 ± 41.5	138.0 ± 125.1	0.468
Pain	172.5 ± 15.0	183.2 ± 56.7	−10.7 ± 28.7	0.711
Confusional state	165.0 ± 21.2	183.0 ± 55.5	−18.0 ± 39.7	0.651
Hematoma	165.0 ± 63.4	183.0 ± 54.9	−18.0 ± 39.7	0.651
Deep infection*	100.0 ± 0.0	184.0 ± 53.9	−84.0 ± 54.4	0.129
Cardiac intercur.*	210.0 ± 42.4	181.2 ± 55.1	28.8 ± 39.6	0.471
**Major complic.**	173.3 ± 70.2	182.9 ± 54.4	−9.6 ± 32.8	0.772
**Total complic.**	190.9 ± 69.6	176.5 ± 41.9	14.4 ± 15.5	0.357

* Major complications.

**Table 7:** Duration of the procedure depending on the occurrence of complications stratified by surgical approach: posterior approach.

Complication	Occurrence of complications		Mean difference ± Standard error of the diff.	p-value
	Yes Mean ± SD	No Mean ± SD		
Superficial infection	252.5 ± 58.5	222.2 ± 61.2	30.3 ± 31.1	0.885
Urinary Intercur.	257.8 ± 56.3	222.0 ± 61.1	35.8 ± 31.1	0.252
Pain	316.4 ± 97.0	216.9 ± 52.9	99.5 ± 22.0	0.000
Confusional state	277.0 ± 94.8	222.3 ± 60.5	54.7 ± 43.5	0.211
Hematoma	235.0 ± 31.2	222.9 ± 61.7	12.1 ± 35.9	0.737
C5 neuropraxia	220.0 ± 78.7	223.4 ± 60.8	−3.4 ± 31.2	0.914
Deep infection*	206.2 ± 33.9	225.0 ± 63.0	−18.8 ± 20.3	0.357
Implant intercur.*	306.7 ± 61.1	220.9 ± 59.7	85.8 ± 35.0	0.016
Cardiac intercur.*	209.3 ± 17.2	223.6 ± 61.8	−14.3 ± 35.9	0.691
Death*	197.5 ± 24.7	223.7 ± 61.5	−26.2 ± 43.7	0.550
**Major complic.**	225.6 ± 53.3	222.9 ± 62.5	2.8 ± 16.6	0.868
**Total complic.**	248.6 ± 70.8	209.9 ± 50.9	38.7 ± 13.0	0.004

* Major complications.

**Table 8:** Duration of the procedure depending on the occurrence of complications stratified by surgical approach: combined approach.

Complication	Occurrence of complications		Mean difference ± Standard error of the diff.	p-value
	Yes Mean ± SD	No Mean ± SD		
Superficial infection	315.0 ± 0.0	357.8 ± 119.1	−42.8 ± 128.7	0.753
Pain	340.0 ± 0.0	353.7 ± 120.3	−13.7 ± 129.9	0.920
C5 neuropraxia	360.0 ± 0.0	350.3 ± 120.4	9.7 ± 130.0	0.944
Implant intercur.*	460.0 ± 0.0	333.7 ± 108.5	126.3 ± 117.2	0.330
New compression*	138.0 ± 0.0	387.3 ± 62.0	−246.3 ± 67.0	0.014
**Major complic.**	299 ± 227.7	372.8 ± 56.8	73.8 ± 163.0	0.726
**Total complic.**	322.6 ± 117.0	424.5 ± 54.4	101.9 ± 89.9	0.308

* Major complications.

## DISCUSSION

Surgical decompression is considered the gold standard procedure for treating and preventing neurological deficits in cervical spondylotic myelopathy (CSM). ^(^ ^10^ Nevertheless, a discussion still remains about the surgical approach for each case, considering the number of addressed segments. Thus, this study aimed to evaluate the early postoperative complications associated with surgical approaches to the cervical spine of individuals with CSM, comparing the anterior surgical, the posterior surgical, and the combined approaches, evaluating the relation between complications and the used approach, number of segments involved in the procedure, and surgical time.

Thus, we conducted a retrospective study based on a database obtained from electronic medical records and imaging exams. All 169 selected patients had undergone a surgical procedure to treat CSM by the same surgical team from 2008 to 2015 in a tertiary hospital. Retrospective studies on databases can lead to information collection errors since we established no previous research protocol. To minimize this bias, we collected data by a complete evaluation of patients’ medical records and nursing staff and the physiotherapy team’s notes.

Several previous studies have evaluated complications resulting from surgery to treat cervical spine conditions. ^(^ ^11^ ^)-(^ ^18^ However, most studies regarding surgical approaches to the cervical spine included patients with different types of diseases, such as tumors, traumatic injuries, herniated disc-associated radiculopathy, spondylodiscitis, and (less commonly) vascular malformations and deformities. ^(^ ^6^ Thus, we believe that selecting a sample of patients with the same disease may reduce the risk of selection bias since the indication for surgical treatment was CSM in all cases.

Likewise, the literature has no consensus regarding the definitions of postoperative complications. Thus, we agree with Campbell et al. ^(^ ^7^ and Fehlings et al. ^(^ ^19^ and follow the same standards as these authors regarding the definition of early complications as an adverse event that occurs within the first 30 days after surgery, ruling out complications after this period (which we considered late complications). Furthermore, even after several previous studies, ^(^ ^20^ ^)-(^ ^23^ controversy remains regarding the severity of complications, making it difficult to use a pre-established pattern. Thus, we once again follow the model used by Campbell et al. ^(^ ^7^ and chose to assess early complications as minor and major, the difference being the need for new surgical intervention or permanent sequelae.

According to Montano et al., ^(^ ^24^ prolonged operative times and increased blood loss are individually associated with an increase in the overall complication rate regardless of whether the approach is anterior, posterior, or combined. ^(^ ^25^ Our results showed no statistically significant difference between the mean surgical time of anterior approach surgeries when we separated patients with complications from cases without them. However, when we evaluated the mean surgical time of procedures performed by the posterior approach, the occurrence of total complications and, specifically, pain and complications with the implant were statistically significant in longer surgeries. In the total research sample, superficial infections (p = 0.014), complications with implants (p = 0.000), and total complications (p = 0.005) were more prevalent in cases of longer surgery.

The anterior approach, involving decompression, followed by arthrodesis, is widely indicated in cases with an anterior compressive component and associated kyphosis. Moreover, it is considered a safe and effective procedure to treat CSM. ^(^ ^26^ The complication with the highest incidence in this approach is dysphagia and one of the most severe complications is airway obstruction, which can have several causes, such as edema in the upper airways and postoperative hematoma. ^(^ ^25^ In our study, the rate of dysphagia in patients who underwent the anterior approach totaled 17.3%. Dysphagia is believed to be related to the extension and duration of esophageal withdrawal or retraction during the surgical procedure due to compromised blood flow to the mucosa. ^(^ ^25^ An information that may explain the higher rate of dysphagia in our study, compared with previous studies, was the use of notes from the nursing and physiotherapy team to obtain the data.

Regarding the posterior surgical approach, Shammassian and Hart^25^ reported a wound infection rate of 4.7% and attributed postoperative immobilization, pressure on the wound, changes in vascular supply, and tension in wound closure as probable causes for this complication. We found a 3.6% rate of superficial infection in cases operated by the posterior approach in our sample. However, this result showed no statistically significant difference (p = 0.072) when we compared the group of patients undergoing surgical treatment in up to two segments with patients undergoing surgical treatment involving three or more spinal segments. On the other hand, we found a statistically significant correlation (p = 0.014) between superficial infection and longer surgical time.

Another aspect we considered significant and previous studies failed to do so^6^ ^),(^ ^7^ was the occurrence of pain in the early postoperative period. We found this minor complication in 7.7% of the patients who underwent the anterior approach and in 6.4% of patients treated using the posterior approach, corresponding to 7.1% of all complications in this study. We believe that many authors have chosen to disregard pain in their assessments of early complications since differentiating the pain symptom expected in the early postoperative period from a pain symptom resulting from a complication is difficult. We defined pain as a complication when the symptom was worse than in the preoperative period to minimize this risk.

This study has some limitations. First, the study design is a retrospective analysis of a database. However, knowing this potential bias in the collection of information, we used the notes of the medical team and those of the nursing and the physiotherapy teams. This fact allowed us to detect complications that we think are more effective. The second limitation was the lack of standardization of a previously established definition of early complication and the subjectivity in dividing minor and major complications. Thus, we chose to use the definition models used by Campbell et al., ^(^ ^7^ which enabled us to define with some ease what would be an early complication and differentiate major complications from minor ones.

However, our study managed to include patients with the same disease (CSM), making our sample more homogeneous. Moreover, the same team performed all surgical procedures, reducing the bias inherent to surgeons’ experience.

## CONCLUSION

Our results agree with findings reported in the literature by showing that dysphagia, pain, and superficial surgical wound infection were the most frequent postoperative complications. However, establishing a statistical relationship between the incidence of complications and the surgical time, surgical approach, and number of fused segments was impossible.
